# Inhibition of astrocytic glycine transporter-1: friend or foe for ameliorating NMDA receptor hypofunction?

**DOI:** 10.3389/fncel.2024.1389718

**Published:** 2024-05-24

**Authors:** Philipp Singer, Benjamin K. Yee

**Affiliations:** ^1^Roche Diagnostics International AG, Rotkreuz, Switzerland; ^2^Department of Rehabilitation Sciences, The Hong Kong Polytechnic University, Kowloon, Hong Kong SAR, China; ^3^Mental Health Research Centre, The Hong Kong Polytechnic University, Kowloon, Hong Kong SAR, China

**Keywords:** antipsychotics, glycine reuptake, glycine-B site, neuron-glial interaction, NMDA receptors, schizophrenia

## Introduction

The glutamate hypofunction hypothesis of schizophrenia (Carlsson and Carlsson, 1990; Olney and Farber, 1995; Olney et al., 1999; Coyle, 2006; Moghaddam and Javitt, 2012; Coyle et al., 2020) has been highly influential in the search of novel drugs for the treatment of negative and cognitive schizophrenia symptoms—which current antipsychotic drugs cannot meet. Both metabotropic (e.g., mGluR1, mGluR5) and ionotropic glutamate receptors (namely, NMDARs) have been targeted (Javitt, 2004; Moghaddam, 2004; Maksymetz et al., 2017; Pei et al., 2021; Dogra and Conn, 2022). Reports that schizophrenia negative and cognitive symptoms could be improved by adjunctive treatment of glycine and sarcosine (Javitt et al., 1994; Heresco-Levy et al., 1996a,b, 1999; Tsai et al., 2004; Lane et al., 2010; Lin et al., 2017) had led to the proliferation of synthetic compounds designed to block the reuptake of glycine via glycine transporters (Harvey and Yee, 2013; Singer et al., 2015). This is predicted to boost glutamatergic signaling at NMDARs and thereby alleviate symptoms according to the glutamate hypofunction hypothesis of schizophrenia. The hypothesis is based on: (i) Occupancy of the glycine (strychnine-insensitive) binding site in the NMDA receptor (also known as glycine-B site), by glycine or D-serine, is required for NMDAR channel activation by glutamate, and (ii) Glycine-B site occupancy is normally maintained at sub-saturating levels by removal of extracellular glycine in the vicinity of the synaptic cleft through active glycine reuptake. Thus, elevation of extracellular glycine by blocking its reuptake should effectively enhance impulse-dependent NMDAR currents. To minimize interference of inhibitory neurotransmission at glycinergic synapses mediated by strychnine-sensitive glycine receptors (Gomeza et al., 2003), drug development has primarily focused on inhibitors specific for glycine transporter 1 (GlyT1) to avoid blockade of glycine transporter 2 (GlyT2) (see Harvey and Yee, 2013). Indeed, there is a noticeable absence of published human studies on GlyT2 inhibitors (Schmidt and Thompson, 2016).

Several synthetic selective GlyT1 inhibitors had displayed promising outcomes in preclinical studies during the 1990s and 2000s, but none of them could advance to the bedside due to poor efficacy in subsequent clinical trials (Singer et al., 2015; Cioffi, 2018; Zakowicz and Pawlak, 2022). Bitopertin, developed by Hoffman-La Roche, had reached phase III trials, following highly encouraging phase II results (Umbricht et al., 2014; Bugarski-Kirola et al., 2017; Kantrowitz et al., 2017; Pinard et al., 2018). However, the multi-center trials had ended with disappointment and termination of the drug's development as a potential new generation of adjunctive antipsychotic medication (Singer et al., 2015; Zakowicz and Pawlak, 2022). At the time of writing, Iclepertin (BI 425809), developed by Boehringer Ingelheim (Fleischhacker et al., 2021; Rosenbrock et al., 2023), remains the only other GlyT1 inhibitor currently being evaluated at phase III (NCT04846868, NCT04846881) as an adjuvant treatment to improve cognitive functioning in schizophrenia. The outcomes of these trials are expected in 2025.

Based on behavioral phenotyping of two mouse lines with conditional GlyT1 disruption, we have previously suggested that the behavioral effects of GlyT1 inhibition are critically dependent on cell type and brain region (Möhler et al., 2011; Singer et al., 2015). We predicted that such dependency could pose a major roadblock in drug development. Divergent antipsychotic and pro-cognitive phenotypes have been reported between mutant mice lacking GlyT1 in forebrain (the cerebral cortex and striatum) neurons and mice lacking GlyT1 in both neurons and astrocytes throughout the telencephalon (Möhler et al., 2011; Singer et al., 2015). Critically, NMDAR currents in the hippocampus were enhanced when GlyT1 was restricted to forebrain neurons (Yee et al., 2006), but not when the deletion was extended to astrocytes (Singer et al., 2009). The additional deletion of glial GlyT1 in the hippocampus apparently nullified the pro-NMDAR effects seen after selective neuronal GlyT1 deletion. A similar impression is also apparent when the behavioral phenotypes between the two mutant mouse lines are compared. These outcomes led us to suggest that the therapeutic potential of systemic, brain wide GlyT1 inhibition would likely be limited and inconsistent. The scant clinical data available had also pointed to an impression of “more means less,” of which the developers of bitopertin were certainly aware. They already emphasized the need for careful dose titration after phase 2 trials and proposed that a moderate level of GlyT1 occupancy at around 50% is desirable for achieving the strongest clinical effect (Umbricht et al., 2014). To this end, efforts have been made to develop radio ligands for personalized dose determination. However, we believe that dose titration alone is not sufficient to mimic the critical cell-type and regional specificity of GlyT1 blockade, which we speculate is also a critical determinant for a GlyT1 blocker's antipsychotic potential—efficacy against both positive and negative symptoms.

Here, we attempt to explain some of the roadblocks above and speculate how the neuropharmacological profile of GlyT1-inhibiting drugs may be critically determined by its concomitant regulation of astrocytic GlyT1 activity. The speculative model takes into account evidence for neuron-glial cross talk in the regulation of the synthesis and tracking of glycine as well as D-serine, the two major endogenous obligatory co-agonists at the glycine-B site of NMDARs.

**Hypothesis 1: – Disruption of the serine shuttle by astrocytic GlyT1 blockade can impair NMDAR signaling**.

**Figure 1:**
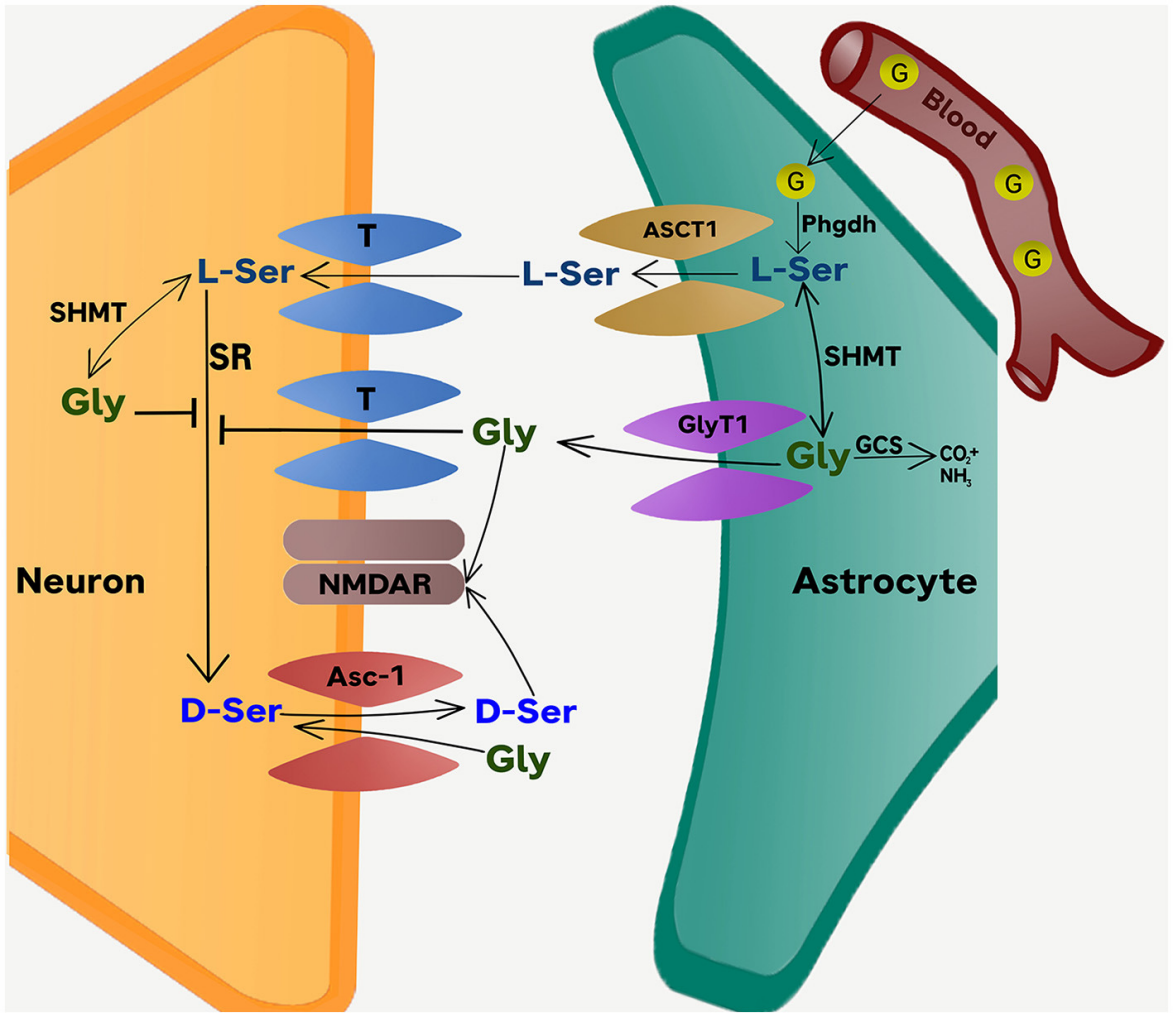
The Phgdh-dependent serine shuttle mechanism. The scheme depicts the conversion of glucose into L-serine in astrocytes and the role of Phgdh-derived L-serine in providing D-serine and glycine to activate synaptic NMDARs. The model also illustrates the dual effect of glycine on D-serine metabolism. The first is the direct inhibition of the neuronal SR by glycine. The second is the transient increase in D-serine release through a Gly/D-Ser exchange catalyzed by the Asc-1 transporter. Serine and glycine are released from astrocytes through ASCT1 and GlyT1 operating in reverse mode (also see Figure 2). Under pharmacological blockade of GlyT1, the primary pool of extracellular glycine is increased as the reuptake of glycine is stopped. In this situation, any additional production of glycine from L-Serine is unlikely to have a significant impact on the already elevated levels of extracellular glycine. At the same time, D-serine which originates from the conversion of L-serine in neurons, becomes a crucial source of D-serine for binding to the glycine-B site. The net effect of GlyT1 inhibition therefore effectively reduces the neuronal production and release of D-serine into the synapse. The disruption in the serine shuttle is expected to undermine, rather than enhance, the excitability of NMDAR at glutamatergic synapses (as determined by brain regions or age), where D-serine acts at the primary co-agonist of NMDAR activation at the glycine-B site. The distribution of NMDAR sites that are more dependent on D-serine than glycine likely varies across brain regions and is modified by other factors such as age and experience. ASCT1, Amino acid transporter (*SLC1A4*); Asc-1 transporter, alanine-serine-cysteine transporter (*SLC7A10*); D-Ser, D-serine; G, glucose; Gly, glycine; GCS, glycine cleavage system, a.k.a. the glycine decarboxylase complex or GDC; GlyT1, Glycine transporter 1 (*SLC6A9*); L-Ser, L-serine; Phgdh, Phosphoglycerate dehydrogenase; SHMT, Serine hydroxymethyltransferase; SR, serine racemase. T, Various types of transporters contributing to the up-take of glycine and L-serine into neurons from the synaptic cleft.

The availability of glycine and D-serine at NMDAR-containing glutamatergic synapses is tightly regulated by the surrounding astrocytes (Snyder and Kim, 2000; Betz et al., 2006; Haydon and Carmignoto, 2006; Wolosker, 2011; Shibasaki et al., 2017). One regulatory mechanism depends on the collaborative metabolic interaction between astrocytes and neurons, known as “serine shuttle” (Wolosker and Radzishevsky, 2013). By altering the equilibrium of glycine and serine metabolism in neurons and astrocytes, inhibition of GlyT1 is expected to interfere with the regulatory function of the serine shuttle as depicted in Figure 1.

Raising extracellular glycine levels has been shown to reduce extracellular D-serine concentration *in vivo* indicating that glycine can modify D-serine metabolism (Neame et al., 2019). On the other hand, blocking glycine reuptake into astrocytes via GlyT1 effectively removes a major source of glycine. To compensate for the ensuing fall in intracellular glycine, the conversion of L-serine to glycine catalyzed by serine hydroxymethyltransferase (SHMT) would rise. The resulting astrocytic L-serine deficit would in turn limit the shuttling of L-serine into neighboring neurons (Figure 1), where L-serine is converted to D-serine and glycine. According to this model, curtailing the L-serine shuttle (astrocytes → neurons) is expected to lower the occupancy of glycine-B sites (at NMDARs) due to a fall in D-serine releasable by neurons into the synaptic cleft. The excitability of NMDARs is therefore predicted to diminish rather than enhance. The impact would be the largest where glycine-B site occupancy is critically determined by the synaptic pool of D-serine, which serves as the primary obligatory co-agonist at the NMDARs and thus can effectively regulate NMDAR excitability.

**Hypothesis 2 – Extracellular release of glycine via astrocytic GlyT1 can positively modulate NMDAR excitability**.

Astrocytes are a major pool of glycine in the brain. Besides glycine re-uptake from extracellular space, another source of astrocytic glycine depends on the conversion of glucose obtained from the blood to L-serine by phosphoglycerate dehydrogenase (Phgdh) and the subsequent conversion of L-serine to glycine by SHMT (which also takes place in neurons) (see Figure 1). In neurons, L-serine is also converted to D-serine by serine racemase (SR). Disruption of this pathway is expected to deprive a major source of releasable D-serine in the synaptic space and therefore reduce glycine-B site occupancy at NMDARs. Yet, NMDAR-mediated signaling appears normal in mice lacking SR with reports of intact NMDAR fast EPSPs and EPSCs (Basu et al., 2009; Li et al., 2013; Rosenberg et al., 2013; Neame et al., 2019). It follows that there is a sufficient baseline level of extracellular glycine supported by alternative mechanisms to maintain near-normal glycine-B site occupancy in the NMDARs of SR-null mice. Moreover, it has been shown that the synthesis of glycine by PHGDH in astrocytes can be critical. Significant impairments in NMDAR-mediated neurotransmission are apparent in SR-null mice when PHGDH activity was suppressed (Neame et al., 2019). It follows that disruption in the release of glycine from astrocytes can influence the excitability of synaptic NMDARs.

It is now known that GlyT1 also mediates the flow of glycine from astrocytes into extracellular space by operating in a *reverse* mode as opposed to its re-uptake mode of operation (Harsing and Matyus, 2013; Shibasaki et al., 2017). Blockade of astrocytic GlyT1 may undermine the release of glycine synthesized by Phgdh inside astrocytes and consequently the excitability of NMDARs. We hypothesize that the functional significance of this glycine source in SR-null mice may be revealed by the specific deletion of astrocytic GlyT1 in these mice, which is predicted to undermine NMDAR excitability, resembling the effect of Phgdh inactivation in SR-null mice (Neame et al., 2019).

**Hypothesis 3 – Inhibiting astrocytic GlyT1 during presynaptic activation can reduce the availability of glycine in the synapse and limit postsynaptic NMDARs excitability**.

**Figure 2:**
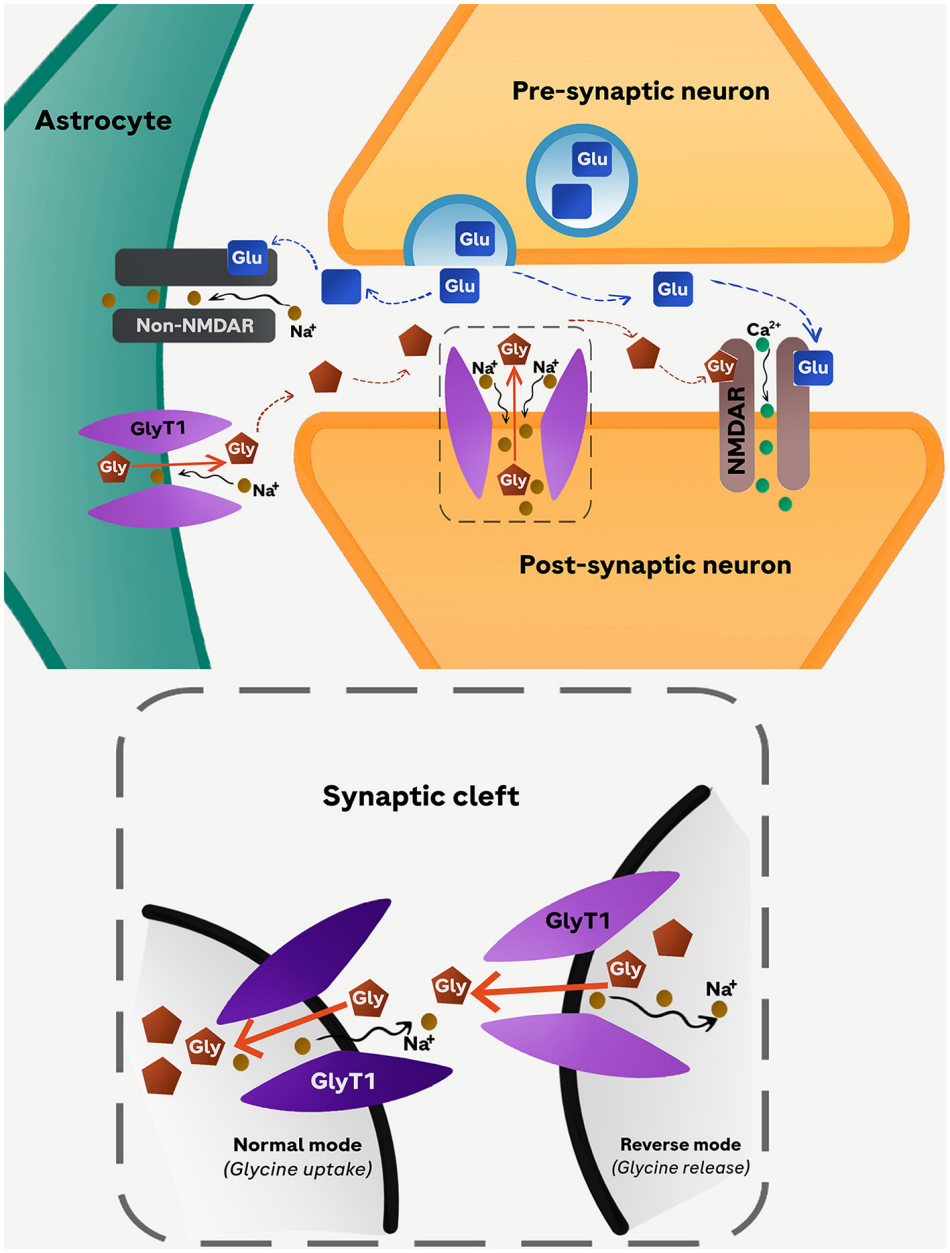
Proposed cyclic operation model of GlyT1. Glutamate released into the synaptic cleft from the presynaptic neuron stimulates non-NMDARs (AMPA/kainate) receptors in astrocytes leading to an influx of Na^+^. The resulting depolarization of the astrocyte membrane triggers the reverse-mode operation of GlyT1 causing an increased glycine efflux from astrocytes into the synaptic cleft. The simultaneous release of glycine (from astrocytes) and glutamate (from presynaptic neuron) activates NMDARs located in the membrane of the postsynaptic neuron. As the concentration of glycine in the synaptic cleft increases further, the direction of GlyT1 operation switches to normal-mode operation reabsorbing glycine back into astrocytes. Glu, glutamate; Gly, glycine.

Harsing and Matyus (2013) were the first to show that GlyT1 in astrocytes operates in a cyclic manner, oscillating between phases of synaptic activation and inactivation, which correspond to the depolarization and repolarization phases of presynaptic glutamatergic axon terminals, respectively (Figure 2). The release of glutamate from the presynaptic terminals triggered by incoming action potentials is capable of activating AMPA/kainite (non-NMDA) receptors expressed in nearby astrocytes. The resulting inward Na^+^ current would switch the operation of astrocytic GlyT1 from its re-uptake mode to the reverse mode. Hence, during active release of glutamate, astrocytic GlyT1 is releasing glycine into, rather than removing it from, the synaptic cleft and therefore promote NMDAR activation.

According to this cyclic model, blockade of astrocytic GlyT1 during presynaptic activation would curtail the elevation of extracellular glycine from the astrocytic pool, although it is expected to elevate ambient extracellular glycine levels at quiescent axonal terminals. In the former scenario, the NMDARs in the postsynaptic active zones would become less responsive to stimulation by glutamate assuming that the glycine-B site of NMDARs is not saturated. Under a global blockade of GlyT1, therefore, the pro-NMDAR action resulting from the blockade of neuronal GlyT1 would be undermined by the concomitant blockade of astrocytic GlyT1. This may in part explain our observations that restricting GlyT1 deletion to neurons could yield more consistent pro-NMDAR phenotypes than extending its deletion to astrocytes (Möhler et al., 2011; Singer et al., 2015).

The full neurophysiological implication of the cyclic model on individual synaptic connections, as well as at the network level, certainly warrant further exploration. The temporal dynamics and the molecular mechanisms governing the switch between the depolarization (reverse mode) and repolarization (reuptake mode) modes of GlyT1 in astrocytes must be empirically verified to allow the formulation of testable hypotheses to be evaluated at the behavioral levels with suitable preclinical models (Singer and Yee, 2015). Appreciating the bidirectional regulation of glycine trafficking by this population of GlyT1 could revitalize research into GlyT1- blockers capable of acting selectively on one or the other mode of operation. Unraveling the molecular switch between GlyT1's two modes of operation may pave the way for their functional distinction permitting a more precise enhancement of NMDAR function in the schizophrenic brain, thereby overcoming a significant roadblock to drug development. It may be conceivable that synthetic drugs and biologics that may slow down or speed up this switching process could be identified. Finally, the possibility that dysregulation of astrocytic GlyT1 may be linked to negative and cognitive symptoms attributed to underactivity of cortical dopamine D1 receptors is further highlighted by the report that dopamine could modulate the release of glycine from cortical astrocytes via GlyT1 (Shibasaki et al., 2017). This may lead to novel GlyT1-based therapeutic strategies to address imbalances in dopaminergic and glutamatergic signaling in schizophrenia.

In conclusion, we contend that any pharmacological strategies aimed at enhancing NMDAR function by increasing synaptic glycine or D-serine levels must accommodate likely concomitant impact on the serine shuttle, which underlines the complex metabolic interplay between glycine and D-serine in terms of synthesis, clearance and trafficking within and across neurons and astrocytes. This in turn critically determines the cyclical operation of GlyT1 in astrocytes and consequently the excitability of NMDAR, as summarized in the three hypotheses presented here. Suffice to say, they have not exhaustively incorporated the full complexity of glycine regulation in the brain, which also depends on a myriad of amino acid transporters and metabolic pathways omitted here. All of which, however, deserve consideration even when a single player, such as GlyT1, is targeted specifically, not to mention the likely adaptive changes that long-term exposure to such drugs inevitably will induce.

## Author contributions

PS: Writing—original draft, Writing—review & editing. BY: Writing—original draft, Writing—review & editing.
